# Developing the Physical Performance in Youth Soccer: Short-Term Effect of Dynamic–Ecological versus Traditional Training Approach for Sub-Elite U13 Players—An Ecological Exploratory Cluster Randomised Trial

**DOI:** 10.3390/jfmk9020083

**Published:** 2024-05-03

**Authors:** Italo Sannicandro, Samuel Agostino, Massimiliano Abate Daga, Franco Veglio, Federico Abate Daga

**Affiliations:** 1 Department of Experimental and Clinical Medicine, University of Foggia, 71121 Foggia, Italy; italo.sannicandro@unifg.it; 2 Department of Medical Sciences, University of Turin, 10124 Turin, Italy; samuel.agostino@unito.it (S.A.); massimiliano.abatedaga@unito.it (M.A.D.); franco.veglio@unito.it (F.V.); 3 Department of Clinical and Biological Sciences, University of Turin, 10124 Turin, Italy

**Keywords:** dynamic–ecological approach, motor performance ability, soccer performance, young soccer player

## Abstract

Currently, research in youth soccer consists of methodological choices that can raise activity volumes and exercise intensity to promote proper training for youth soccer demands. Therefore, the present cluster randomised trial aims to evaluate the effects of the dynamic–ecological approach on the physical performance parameters compared with a traditional one in a group of sub-elite U13 players. Thirty-five male children (age 12.16 ± 0.55 years; weight 45.59 ± 7.15 kg; height 145.5 ± 4.2 cm; BMI 15.8 ± 2.1 kg·m^−2^) were recruited for this trial from two teams belonging to sub-elite soccer schools and randomly assigned to a dynamic–ecological approach (DEA) or a traditional training (TTG) group. The training program lasted six weeks and consisted of 18 training sessions of 90 min each (3 sessions per week). The sample was evaluated by the standing long jump (SLJ), hop test (HT), 10 m sprint (10 m), 10 × 5 m shuttle run test (SRT), and leg raise test (LR). The DEA group showed significantly higher results in the SLJ (*p* < 0.001), HT left leg (*p* < 0.001), 10 m sprint (*p* < 0.001), and SRT (*p* < 0.001). In conclusion, the dynamic–ecological approach provides higher performance adaptations. Therefore, this approach can be considered a suitable method to optimise pre-pubertal player training, mainly when no fitness or strength coach is available.

## 1. Introduction

Youth sports represent a common experience for thousands of children worldwide. They approach different sports disciplines for various reasons, and soccer is one of them. Soccer is one of the most practised sports due to its elevated adaptability to spaces and multiple players, simplicity of rules, and low-cost participation. It is estimated that millions of children and adolescents are playing soccer worldwide [1]. Notably, youth football is characterised by skill acquisition and technical development over competitive outcomes. Therefore, young players’ training schedules must focus on teaching fundamentals such as dribbling, passing, shooting, and ball control, laying a solid foundation for players’ future growth. Secondly, soccer demands high physical efficiency and appropriate motor performance abilities to succeed in senior and youth competitions [2,3]. Therefore, relevant programs for physical efficiency and motor coordination development should be set up to sustain the physical demands of the discipline. Despite this, an unstoppable decline in motor competence across generations contrasts with the need for sufficient motor skills in sports disciplines, and this trend prompts reflection on the role of youth sports training in the physical development of children and adolescents [1,2,3].

Youth sports training should focus on technical and methodological aspects, especially for beginners with lower motor performance abilities. This approach establishes a solid foundation by addressing individual needs and improving overall skill development in young athletes, which will benefit them in their future sports careers. However, despite the importance of solid motor competence in succeeding in sports competitions, the current literature needs to include this decline in motor proficiency in children and adolescents [4]. Sedentary lifestyles; increased screen time and engagement in passive activities like video games; environmental changes; a reduction in outdoor play; overreliance on technology; changes in family dynamics; and, last but not least, cutbacks in school physical education programs and limited access to sports and recreational activities contribute to this decline [4,5,6,7,8,9]. Furthermore, most training shows limitations and rarely considers children’s motor abilities and background. A lack of training customisation based on current motor abilities, short-term gains focalisation, and the trend for trainers to be rooted in old and established teaching methods are only some limitations in sub-elite sports training [10].

Therefore, to fight against reducing children’s motor skills caused by decreased daily physical activity levels, physical education and sports-specific training have adopted different methodological and organisational purposes [5,11,12] to improve activity volumes, exercise intensity, and teaching styles [13,14]. One of these possible strategies has been identified as the dynamic–ecological approach. This method emphasises the interconnectedness between players, their environment, and the game. In addition, it considers how these elements interact and influence each other in real-time situations. Therefore, considering its insight, the dynamic–ecological approach naturally leads coaches and players to adapt their strategies and behaviours to optimise performance by understanding the dynamic relationships within the team and with the external environment [15,16,17].

On the contrary, the traditional training approach to team sports typically involves structured and disciplined training methods, focusing on established techniques, strategies, and conditioning routines. This approach often emphasises fixed drills and predefined patterns of play to enhance technical skills, tactical understanding, and physical conditioning among team members. Traditional training methodologies may prioritise repetition and adherence to predetermined plans, aiming for consistency and mastery of specific skills [15,18]. This approach has been widely employed and proven effective in fostering fundamental abilities. Nevertheless, it might need more adaptability and context-specific considerations identified in more modern ecological dynamic approaches to training for team sports [14,19]. Hence, based on existing literature, a dynamic–ecological approach prioritises motor learning, technical proficiency, adaptation, the transferability of skills to diverse contexts, and a self-regulated understanding of fundamental tactical concepts in sports. This approach’s strength is guiding players to use their natural movement tendencies to develop coordination, where variability becomes helpful in creating smooth and seamless motor patterns. Several studies have shown that dynamic–ecological approaches can improve decision-making, anticipation, and perceptual skills compared to traditional methods [20]. Furthermore, the dynamic–ecological approach often results in a better transfer of skills to actual performance settings than conventional training methods [21]. Thus, this approach is relevant in young soccer player training because it requires environment exploration and problem-solving motor/cognitive activity, increases motor engagement time, and allows for high exercise intensity [22,23,24]. For this reason, it has been deeply adopted in recent years to acquire more versatile motor skills, especially in youth soccer [15,16]. 

Furthermore, these characteristics can be vital for a young player during talent selection and discrimination. The talented player benefits from practising in variable environments, which helps them explore and find many effective solutions in their sport. By keeping information and movement together, they can better find solutions despite the complexity of the environment [14]. In addition, coaches and talent scouts usually evaluate players during matches, the highest expression of a dynamic–ecological climate in soccer, and their subjective perception is still highly considered when scouting and discriminating soccer talent [25,26,27]. 

Despite the widespread adoption of this new methodological approach in youth soccer and the acknowledgement of its potential benefits, there remains a gap in the literature regarding its effects on developing motor performance abilities in young players. Even though motor skills and physical development are as crucial as technical and tactical abilities for soccer players, research in this area still needs to be conducted [18,28,29]. Therefore, this study aims to verify the effects of a soccer training program based on the dynamic–ecological approach on U13 sub-elite players’ physical efficiency by analysing explosive strength, speed, resistance to speed, and muscle flexibility. This study hypothesises that the dynamic–ecological approach can consistently help players develop adequate physical efficiency and motor coordination. 

## 2. Materials and Methods

### 2.1. The Study Design

This cluster randomised trial was approved by the local government of the Italian Football Federation (FIGC) and by the bioethical committee of the University of Turin (protocol number 0433611). Furthermore, it was conducted according to the Declaration of Helsinki, the CONSORT recommendations for non-pharmacological trials [29], and the American Psychological Association recommendation for research and publication [30]. This study is registered at Clinicaltrials.gov. The clinical registration number is NCT06389227. 

### 2.2. Participants

**Figure 1:**
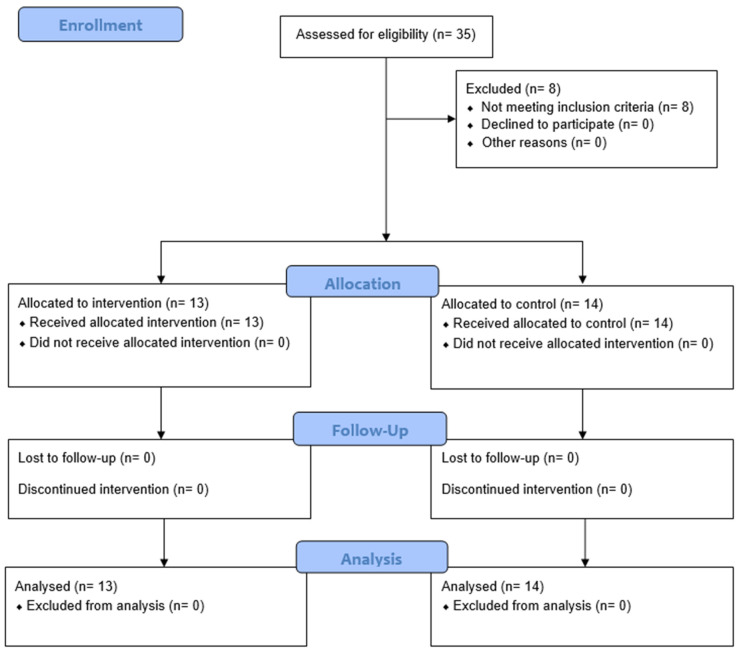
A CONSORT diagram showing the participants’ progress through the trial.

Thirty-five male pre-pubertal children were recruited from two sub-elite soccer schools and randomly assigned by cluster to a dynamic–ecological approach (DEA) or a traditional training (TTG) group (Figure 1). All young soccer players who were included in the study:

(a) Had not suffered an injury in the previous 6 months;
(b) In the 4 weeks before the study, had systematically performed the training sessions without interruptions or absences;
(c) Upon medical evaluation, fell within stages 3 and 4 of Tanner’s classification [31].

All participants were informed about the study, and their parents provided informed consent. An evaluator blinded to the treatment groups performed the physical examination. Performance parameters were collected at baseline after randomisation and at the end of the experimental phase by the same blinded evaluator. The training program lasted six weeks and consisted of 18 training sessions of 90 min each (3 sessions a week). The duration of the study arose from the competitive calendar established by the FIGC, which provided for a suspension of competitive activity after the first six weeks of the youth championship. 

Each young player received a volume of 1620 min of training and 260 min of competition.

Only infield players and uninjured players with at least three years of experience in soccer who performed at least 70% of the programmed schedules (12 of 18) were eligible for the study. Goalkeepers and players who had been injured in the previous six months or did not meet the established minimum compliance were excluded from this study.

Before the study started, parents, trainers, and participants were fully informed about all training and testing procedures and any possible risks and discomforts. In addition, all players were asked not to modify their dietary habits or adhere to extra physical activity or training programs scheduled for this research during the entire study, and they accepted the conditions.

### 2.3. Procedures

The sample was randomly assigned to dynamic–ecological (13 players) or traditional training (14 players) groups. Each group had to include at least one player representing each position (one forward, one midfielder, etc.). Players were measured at baseline and the end of the intervention (6 weeks). All measurements were conducted on the same artificial turf on two separate days to avoid incoming fatigue and irregular surfaces that might affect the measurements. Baseline tests were performed at the beginning of September, while the final evaluations occurred during the second week of October. Three certified fitness and conditioning coaches, supervised by the research team, conducted both testing sessions on sunny days.

The evaluation tests used in this research were chosen consistently with other studies involving young soccer players [32,33]. Lower limb power was evaluated using the standing long jump and the hop (left and right) test. Sprinting ability was assessed with the 10-m sprint test, and resistance to speed was assessed with the 10 × 5 shuttle run. Finally, hamstring flexibility was assessed using the leg raise (left and right) test, measuring the hip range of motion with an angular goniometer. Three minutes of rest were guaranteed between trials to ensure a complete recovery. The standing long jump, hop test (left and right), and 10 m sprint were performed three times, taking the best score. Conversely, the 10 × 5 shuttle run test and the leg raise (left and right) test were performed just once. 

### 2.4. Training Protocols

The DEA completed 18 ninety-minute sessions (3 per week) oriented to soccer training using the dynamic–ecological approach for six weeks. 

The TTG followed a traditional soccer training program for the same period and number of sessions without any intervention attributable to the dynamic–ecological approach (Table 1). The activities assigned to the DEA group were derived from the literature, which includes numerous studies that have described and proposed training through the dynamic–ecological approach and small-sided games [24,34,35], adapting training volumes to the participants’ ages [11,13,16,17].

The programs assigned to the two groups were developed by three experienced coaches with more than 20 years of experience who had previously adopted and monitored the different exercises with other peer groups through Borg scales to understand the feasibility of young player scheduling [36].

During the period under consideration, both groups participated in the same number of matches (n = 6) in the 9 vs. 9 format on a small field according to Italian Football Federation (FIGC) rules and on a small field according to FIGC regulations.

### 2.5. Anthropometrics

Body mass was measured to the nearest 0.1 kg (Rowenta BS1060, Erbach, Germany), with the participants wearing only underwear. Standing height was calculated using a wall stadiometer with a precision of 0.01 m and a 60 to 210 cm range (Lanzoni D01602H, Bologna, Italy). BMI was calculated using a Microsoft Excel sheet (Redmond, WA, USA), where the BMI formula had previously been inserted (BMI = Body weight/(height × height)).

### 2.6. Standing Long Jump Test

The players positioned themselves behind the court’s white back line with feet apart, executing a two-foot take-off while swinging their arms and bending their knees to generate forward momentum. They were directed to leap as far as they could, landing on both feet without tipping backwards. Three tries were allowed, and the highest score among these attempts was considered for evaluation.

### 2.7. Hop Left and Right Test

In this evaluation, the player had to achieve the longest distance possible in a one-legged jump while maintaining balance and ensuring a stable landing. The starting point was assessed behind the white back line, and the final distance was measured from the start line to the heel of the landing leg. Three tries were allowed, and the highest score among these attempts was considered for evaluation.

### 2.8. 10 × 5 Shuttle Run

The player had to complete ten rounds of 5 m shuttle sprints, reversing direction at each return to the starting line. A coach initiated the sprints by lowering an arm while saying “GO” and timing it using a precise manual chronometer (HS-3V-1RET Casio, Japan) capable of measuring in 0.01 s intervals. This process was conducted once, and the recorded times were gathered for evaluation. 

### 2.9. Leg Raise (Left and Right) Test

The leg raise test for flexibility measures players’ hamstring and lower back flexibility. The footballers lay supine on a physical therapy bed with their legs straight. One leg was gently lifted while keeping the knee extended until discomfort or tension was felt. The angle of elevation was noted with an angular goniometer. This test was bilateral and repeated only once for each leg.

### 2.10. Statistical Analysis

All data were cleaned and checked for accuracy. The data distribution was evaluated using histograms and Shapiro–Wilk tests and was found to violate the assumptions for parametric testing; therefore, non-parametric data were analysed using the Mann–Whitney U test and Wilcoxon signed-rank test. Per-protocol analysis was used to estimate the effect of the intervention. Six individuals who agreed to participate in the study programme did not complete their baseline assessment.

Another two participants sustained injuries the day after cluster randomisation (Figure 1). Non-parametric tests evaluated anthropometrics and baseline assessment differences between the experimental and control groups. Even if the groups were randomised by cluster, baseline data analysis may have revealed significant differences between groups in different variables. However, it could be a coincidence, even with randomisation. Nevertheless, post-intervention analysis adjusted for the baseline differences to prevent the regression’s influence on the mean. This adjustment did not change the significance of the baseline difference, supporting the validity of the results after the intervention. Therefore, it was decided to maintain the post-results difference using the Mann–Whitney test to ensure a uniform format. In addition, valuable insights into the temporal effectiveness could be gained by analysing the interaction effect of the dynamic–ecological approach compared to the traditional one. This analysis will provide a deeper understanding of how and when performance improvements differ between the training methods. Therefore, a 2 × 2 ANOVA was incorporated into our analysis, and the results are presented alongside the existing statistical methods.

In addition, to allow for comparison of these outcomes with those of other studies, the data are presented as mean  ±  standard deviation. Data were analysed using SPSS Version 28 (Armonk, NY, USA, 2017).

## 3. Results

### 3.1. Baseline Characteristics

Thirty-five young soccer players were recruited for this study. However, eight were excluded from the calculation because they did not meet the inclusion criteria. In particular, six were goalkeepers, and two were injured at the beginning of the experimental phase. Thus, they could not be considered for this study. Therefore, 27 infield players remained (chronological age 12.16 ± 0.55; weight 45.59 ± 7.15 kg; height 145.5 ± 4.2 cm; BMI 15.8 ± 2.1 years of experience 6.3 ± 1.1). Fourteen (14) players belonged to the dynamic–ecological approach group (DEA), and thirteen (13) to the traditional training group (TTG).

Basic demographic information about the soccer players at baseline shows that there were no significant differences between the two groups for any of the measured variables, as indicated by the *p*-values (Table 2).

Regarding the outcome variables, we observed a significant difference in the 10 × 5 shuttle run test and the hop left-leg test at baseline. These differences could potentially be due to chance, despite randomisation. Corrections were made during the comparison of post-tests to avoid the phenomenon of regression toward the mean. Specifically, the DEA group performed five-tenths of a second faster than the TTG group in the shuttle run test (19.1 ± 0.2 s vs. 18.63 ± 0.6 s, *p* = 0.015) (Table 3). As for the hop left-leg test, the results were 102.8 ± 4.6 cm for TTG and 108.0 ± 8.6 cm for DEA (*p* = 0.026). Since the two sample groups were randomised, these differences may be casual.

### 3.2. Changes in Outcome Measurements

The standing long jump showed significantly higher results (*p* < 0.001) for the DEA group (191.0 cm ± 6.6) compared to the TTG group (166.5 cm ± 5.9). The same trend was noted in the hop test (hop left leg and hop right leg), where the DEA group performed significantly better than the TTG group (139.1 cm ± 15.0 vs. 111.7 cm ± 9.8; *p* < 0.001) in terms of left leg performance. Similarly, the right leg performance was, again, better in DEA than TTG (141.5 cm ± 9.2 vs. 109.6 cm ± 6.1; *p* < 0.001). Furthermore, the 10 m sprint time showed significant improvements (*p* < 0.001) in DEA (1.9 s ± 0.1) compared to TTG (2.2 s ± 0.1). Also, in the 10 × 5 shuttle run test, DEA slightly overperformed (*p* < 00.001), with an average score of 17.5 ± 0.4 s, while TTG scored somewhat higher, at 18.4 ± 0.7 s. 

**Figure 2:**
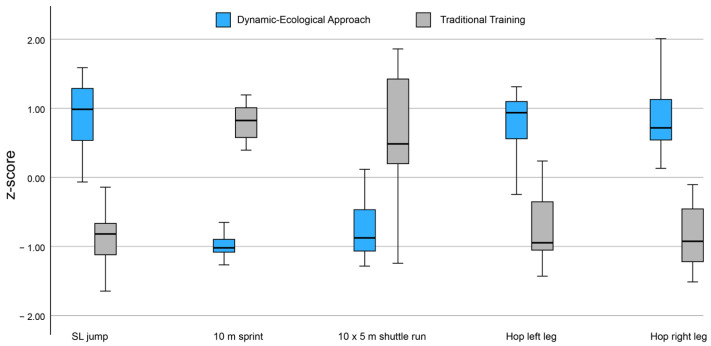
Graphical representation of DEA (light blue boxes) and TTG (grey boxes) in all physical tests that showed significant differences (*p* < 0.05) after the intervention. DEA: Dynamic-Ecological Approach; TTG: Traditional Training Group; SL: Standing Long.

Finally, the leg raise test (leg raise left and leg raise right) was not statistically significant between groups (*p* = 0.233 for leg raise left and *p* = 0.112 for leg raise right), and no differences were detected. Finally, the 10 × 5 shuttle run and hop left-leg test significantly differed at baseline in favour of the TTG. However, there were significant differences in the follow-up in favour of DEA. Therefore, it was decided not to correct the inference in the follow-up results (Table 3) (Figure 2). Furthermore, a verification of temporal interactions was carried out to provide a more complete perspective. A two-way ANOVA was performed to analyse the effects of the measurement time (pre- and post-evaluation) and the training group (dynamic–ecological vs. traditional) for all seven physical tests. 

**Figure 3:**
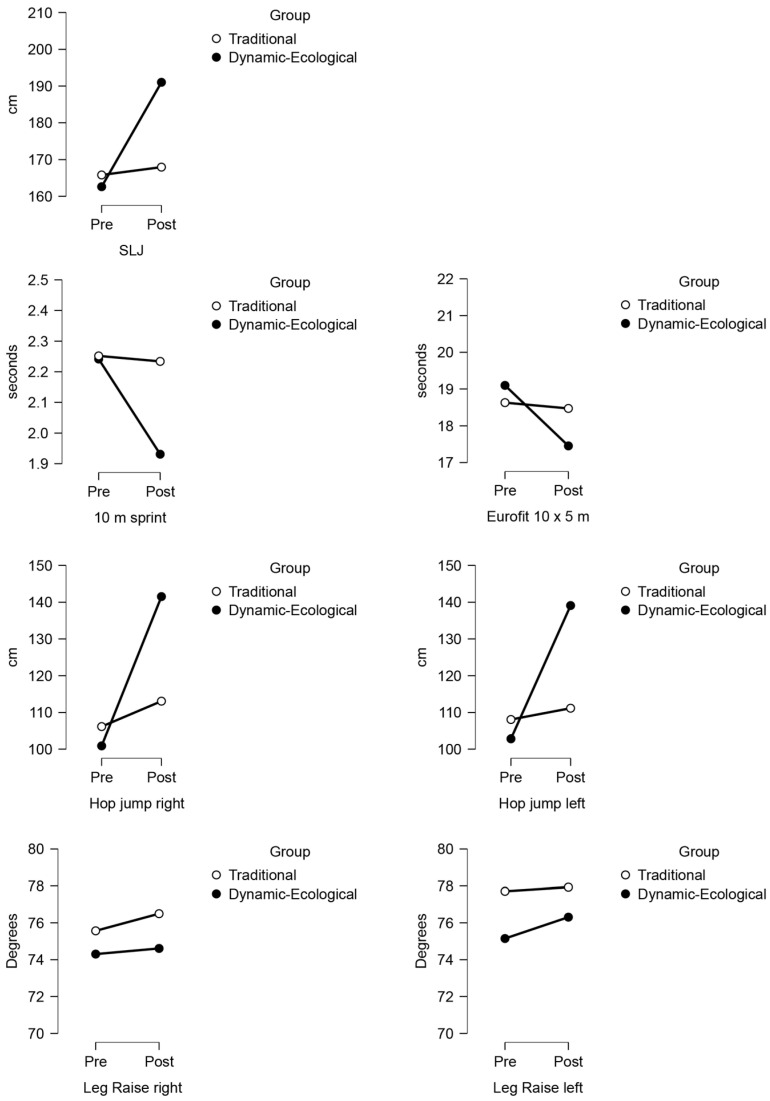
Graphical representation of ANOVA 2 × 2. SLJ: Standing Long Jump.

A two-way ANOVA examined the interaction between exercise and performance in terms of various measures. The results revealed significant interactions for the SLJ (F(1,25) = 115.26, *p* < 0.001), 10 m sprint (F(1,25) = 142.69, *p* < 0.001), Eurofit 10 × 5 (F(1,25) = 47.65, *p* < 0.001), hop left (F(1,25) = 52.72, *p* < 0.001), and hop right (F(1,25) = 122.74, *p* < 0.001) tests. These findings suggest that exercise’s effect on performance differed for these measures. In contrast, no significant interactions were found for the leg raise left (F(1,25) = 0.46, *p* > 0.05) or leg raise right (F(1,25) = 0.24, *p* = 0.63) tests, indicating that the effect of exercise on performance was similar for these measures (Figure 3).

## 4. Discussion

This study aimed to verify the effects of the dynamic–ecological approach on U13 sub-elite players’ physical efficiency by analysing explosive strength, speed, resistance to speed, and muscle flexibility. In addition, this research aspires to contribute to the current literature by exploring and quantifying the benefits of applying the dynamic–ecological approach in youth soccer to enhance motor performance abilities. While prior studies have suggested the benefits of this approach in motor learning across various sports [37,38], there is limited literature regarding its effects on motor skill development and exercise intensity [34]. This study is the first attempt to understand the relationship between dynamic–ecological methods and physical performance factors in young soccer players. Ecological dynamics have been proposed to comprehend how functional movement skills are learned and acquired and to create enriched environments that foster lifelong physical activity engagement [39]. This perspective emphasises integrating physical, cognitive, emotional, and perceptual skills in motor learning [19]. However, only a limited number of studies demonstrating this integrated development are available, especially those concerning motor skill evolution in sports and, particularly, in youth soccer. 

Data from this study indicate that the dynamic–ecological approach increased horizontal explosive strength in standing long jumps by approximately 14%, with a high effect size of 0.85. Similar improvements were observed in single-leg jumps for the left (around 35.4% increase, r = 0.76) and right legs (approximately 40.2% increase, r = 0.83). These outcomes were also better than those achieved through specific strength training sessions in the same age category [40,41]. Thus, these outcomes confirm that the dynamic-ecological dynamic–ecological approach may be a suitable solution to provide significant advantages for U13 sub-elite players, especially when a certified strength training coach is unavailable, and the head coach should administrate the entire schedule. In addition, the sprint ability showed an increase of 13.9%, with a high effect size of 0.85, for DEA. Furthermore, the 10 × 5 m shuttle run demonstrated an 8.7% performance increase in DEA, with a high effect size of 0.71. These results align with other studies that have observed significant performance gains in speed and resistance to speed through game-based soccer training for both post-pubertal [35] and pre-pubertal players [18], employing similar ecological dynamic approaches. These results also align with studies that coincide with findings which have observed the effects on the sprint and shuttle running performance of high-intensity soccer-specific exercises, such as small-sided games [36]. 

Conversely, the dynamic–ecological approach did not significantly impact players’ flexibility. This physical quality requires analytical motor tasks rather than global ones in order to be developed appropriately. For this reason, this approach did not show significant effects on muscle flexibility. In addition, a recent study reported that the player’s biological maturation and age strongly influence flexibility [29]. The authors observed a significant decrease in hamstring flexibility across age categories in a sample of over 600 pre-pubertal soccer players belonging to different age categories from U8 to U12, similar to the sample enrolled for this study. Thus, muscle flexibility is a physical quality that needs strictly focused training to be improved. 

The traditional training group (TTG) showed no statistically significant differences in the comparison between the pre- and post-test periods. This suggests that traditional soccer training in sub-elite U13 players might not effectively stimulate the monitored motor performance abilities in the short term. This fact can be explained by considering the insight of the two different approaches and their administration. The sub-elite level in this age stage is characterised by a middle-to-low level of both players and coaches (the best ones are usually taken by professional clubs); a middle-to-low training load exposure (usually two or three training sessions a week); and a reduced staff size, where a fitness and conditioning coach is often not guaranteed. Therefore, in this scenario, the insight properties of the dynamic–ecological approach may expose players to a higher workload during training sessions, resulting in higher efficiency [42]. For these reasons, this approach may provide more significant benefits to pre-pubertal soccer players, and this study confirms this. Thus, these outcomes suggest a review of youth soccer training organisations and approaches that better stimulate the motor performance abilities of pre-pubertal soccer players [43,44]. Finally, for sub-elite U13 players, the dynamic–ecological approach may be the best choice because of its insight. Dynamic–interactive scenarios, guided discovery, and free exploration tasks require young soccer players to maintain high exercise intensities, which can highly impact players’ physical efficiency. Considering the outcomes of this study and the sample composed of young soccer players, it may be affirmed that the ecological approach significantly increases specific functional motor abilities needed in soccer, such as explosive strength, acceleration, and agility, which are relevant factors for soccer performance [28] Furthermore, the coaching style and method choice impact the process of learning a sport’s technique and influence motor skills [15,16,22,23,24]. This aspect, rarely emphasised in a sport’s technique, but explored in the literature, emerges from studies.

Lastly, the coach’s demands influence the quality of youth soccer performance. This includes the environment that the coach creates, the way in which he or she interacts with young players, and the support he or she provides for solving motor and tactical problems. These are key aspects of the benefit provided by this methodological approach to young players’ development.

In conclusion, this study shows some strengths and limitations. A limitation of this research lies in the need for more monitoring of the adequate active time of each player during training. This fact and the missing measures of the difference in effective intensity resulting from the two methodological approaches (ecological vs. traditional) might have influenced the final results. Secondly, the variables utilised in this study are not “ecological” because, as field tests, they are neither specific nor contextualised in the dynamic–ecological scenario. However, considering the sub-elite scenario, these field tests are “costless” and can be adopted by everybody in each condition. Thus, this can be a strength for the applied research aspect of this manuscript.

Furthermore, the study did not consider the training age of the participants. This characteristic could be a variable that could determine differences in exercise intensity and, thus, differences in assessed physical performance. Conversely, a strength of this study is the period of the experimental phase. This study was conducted during the regular season (competitive period). For this reason, the study holds significant ecological value, as it provides information rapidly applicable to training practice. Finally, future studies should investigate and quantify the time each young player spends in matches, because the intensity of competitive events is maximal and could reveal interesting effects on strength, linear speed, and directional change abilities. Future studies should investigate the impact of the dynamic ecological approach in women’s youth soccer to understand any similarities or differences in the physical performance of young sportsmen and women.

## 5. Conclusions

Compared to traditional training, the dynamic–ecological approach significantly improves U13 sub-elite soccer players’ physical efficiency and motor coordination. The outcome of this study suggests that this methodology can be an efficient approach to guarantee adequate improvement of physical efficiency in U13 sub-elite players. Therefore, coaches and soccer school managers are encouraged to delve into this methodology when organising training schedules. In the future, more research should explore the effect of the dynamic–ecological approach on players’ physical efficiency across different ages and genders, as well as the skill and qualification levels of footballers. This will help coaches and soccer school staff to better understand the influence of this approach on physical conditioning in order to develop young soccer players more effectively. 

## Figures and Tables

**Table 1:** Training protocols in both groups during the experimental phase.

Group	Warm-Up (15 min)	Technical Drills Training (20 min)	Motor Performance Abilities Training (20 min)	Tactical and Situational Abilities (25 min)
DEA Tasks	Movement exploration and technical skills development in autonomy; guided discovery of movements	Functional technical skills development alternating with small-sided games (2 vs. 2, 3 vs. 3, 4 vs. 4, 5 vs. 5)	Traditional games adapted to soccer or pre-sport games, high-intensity point games, invasion or territorial-like games, and decision-making games	Small-sided games and games planned for the age category, 9 vs. 9 (2 × 12 min)
DEA load	Proposed exploration tasks with a 1:0.5 ratio (W:R)	Ratio 1:0.5 (W:R)	Ratio 1:0.5 (W:R)	2 × 12 min, R: 3 min
TTG tasks	Analytical and technical skills development and activation with motor pathways	Analytical and technical skills were developed in individuals, pairs, and stations, such as squares, rhombuses, and pentagons.	Speed drills, quickness of feet with or without tools, balance tasks, joint stability tasks in pairs or play/fun form, multi-jump, etc.	Small-sided games and games planned for the age category, 9 vs. 9 (2 × 12 min)
TTG load	3 × 15 reps for every exercise: 6 analytical exercises	3 × 15 reps for every exercise: 6–8 analytical exercises, ratio 1:0.5 (W:R)	3 × 8 reps for every exercise: 8–10 analytical exercises, ratio 1:0.5 (W:R)	2 × 12 min, R: 3 min

DEA: Dynamic-Ecological Approach; TTG: Traditional Training Group; W: workout; R: resting time.

**Table 2:** Demographic characteristics of the whole sample at baseline. In all anthropometrics, no differences were detected among groups at the baseline.

Variables, Mean (SD), Total (n = 27)	Total (n = 27)	Traditional Training (n = 14)	Dynamic-Ecological Approach (n = 13)	*p* ^a^
Age in years	12.3 (0.5)	12.3 (0.5)	12.3 (0.5)	0.927
Weight (kg)	45.8 (6.7)	46.8 (7.5)	44.7 (5.9)	0.685
Height (cm)	145.2 (3.8)	144.9 (5.2)	145.5 (1.6)	0.611
Training experience	6.2 (0.9)	6.2 (1.1)	6.3 (0.7)	0.544
BMI (kg·m^−2^)	15.8 (2.1)	16.1 (2.2)	15.3 (2.0)	0.65

^a^  *p* values of group comparisons refer to Mann–Whitney U tests for continuous variables. BMI: Body Mass Index; SD: Standard Deviation.

**Table 3:** Comparison of both groups. DEA registered significant improvements in all performance parameters (*p* < 0.001) in the post-test comparison except for the left and right leg raise tests, where no differences were detected among groups.

Variable	Traditional Training (n = 14)		Dynamic–Ecological Approach (n = 13)		
	Pre-Test (Mean ± SD)	Post-Test (Mean ± SD)	Pre-Test (Mean ± SD)	Post-Test (Mean ± SD)	Effect Size r
SLJ (cm)	165.8 ± 5.19	**166.5 ± 5.9**	162.6 ± 4.7	191.0 ± 6.6 *	0.85
10 m sprint (s)	2.25 ± 0.9	**2.2 ± 0.1**	2.2 ± 0.6	1.9 ± 0.1 *	0.85
10 × 5 shuttle run (s)	18.63 ± 0.6	**18.4 ± 01.7**	19.1 ± 0.2	17.5 ± 0.4 *	0.71
Hop left leg (cm)	108.0 ± 8.6	**111.7 ± 9.8**	102.8 ± 4.6	139.1 ± 15.0 *	0.76
Hop right leg (cm)	106.1 ± 9.5	**109.6 ± 6.1**	100.9 ± 4.6	141.5 ± 9.2 *	0.83
Leg raise left (deg)	77.7 ± 3.4	77.7 ± 2.8	75.2 ± 3.5	76.3 ± 2.8	0.32
Leg raise right (deg)	75.5 ± 3.8	76.1 ± 3.1	74.3 ± 3.3	74.6 ± 2.7	0.34

* *p* < 0.001, a significant difference is considered between the TTG post-test and the DEA post-test. Significant differences are highlighted in bold. DEA: Dynamic-Ecological Approach; TTG: Traditional Training Group; SD: Standard Deviation; SLJ: Standing Long Jump.

## Data Availability

The data presented in this study are available upon request from the corresponding author. Due to club owner restrictions, they are not publicly available.
